# Changes in global gene expression of *Vibrio parahaemolyticus* induced by cold- and heat-stress

**DOI:** 10.1186/s12866-015-0565-7

**Published:** 2015-10-23

**Authors:** Sara Urmersbach, Tommi Aho, Thomas Alter, Syeda Sakira Hassan, Reija Autio, Stephan Huehn

**Affiliations:** Institute of Food Hygiene, Freie Universität Berlin, Berlin, Germany; Department of Chemistry and Bioengineering, Tampere University of Technology, Tampere, Finland; Department of Signal Processing, Tampere University of Technology, Tampere, Finland; School of Health Sciences, University of Tampere, Tampere, Finland

**Keywords:** *Vibrio parahaemolyticus*, Gene expression, Thermal shock

## Abstract

**Background:**

*Vibrio* (*V*.) *parahaemolyticus* causes seafood-borne gastro-intestinal bacterial infections in humans worldwide. It is widely found in marine environments and is isolated frequently from seawater, estuarine waters, sediments and raw or insufficiently cooked seafood. Throughout the food chain, *V. parahaemolyticus* encounters different temperature conditions that might alter metabolism and pathogenicity of the bacterium. In this study, we performed gene expression profiling of *V. parahaemolyticus* RIMD 2210633 after exposure to 4, 15, 20, 37 and 42 °C to describe the cold and heat shock response.

**Methods:**

Gene expression profiles of *V. parahaemolyticus* RIMD 2210633 after exposure to 4, 15, 20, 37 and 42 °C were investigated via microarray. Gene expression values and RT-qPCR experiments were compared by plotting the log2 values. Moreover, volcano plots of microarray data were calculated to visualize the distribution of differentially expressed genes at individual temperatures and to assess hybridization qualities and comparability of data. Finally, enriched terms were searched in annotations as well as functional-related gene categories using the Database for Annotation, Visualization and Integrated Discovery.

**Results:**

Analysis of 37 °C normalised transcriptomics data resulted in differential expression of 19 genes at 20 °C, 193 genes at 4 °C, 625 genes at 42 °C and 638 genes at 15 °C. Thus, the largest number of significantly expressed genes was observed at 15 and 42 °C with 13.3 and 13 %, respectively. Genes of many functional categories were highly regulated even at lower temperatures. Virulence associated genes (*tdh*1, *tdh*2, *tox*R, *tox*S, *vop*C, T6SS-1, T6SS-2) remained mostly unaffected by heat or cold stress.

**Conclusion:**

Along with folding and temperature shock depending systems, an overall temperature-dependent regulation of expression could be shown. Particularly the energy metabolism was affected by changed temperatures. Whole-genome gene expression studies of food related pathogens such as *V. parahaemolyticus* reveal how these pathogens react to stress impacts to predict its behaviour under conditions like storage and transport.

**Electronic supplementary material:**

The online version of this article (doi:10.1186/s12866-015-0565-7) contains supplementary material, which is available to authorized users.

## Background

*Vibrio* (*V*.) *parahaemolyticus* is one of the causes of seafood-borne gastro-intestinal infections in humans worldwide [1]. It is widely found in marine environments and is isolated frequently from seawater, estuarine waters, sediments and raw or insufficiently cooked seafood (e.g. shrimp or bivalve molluscs) [2–4]. Consumption of or contact to raw or undercooked seafood containing *V. parahaemolyticus* in relevant numbers, might lead to human infections, mostly associated with gastroenteritis [5, 6].

Different studies investigated the behaviour of *V. parahaemolyticus* under environmental stresses on the phenotypic level (e.g. cold shock, heat shock, high salt concentrations or bile supplementation) [7–9]. Nonetheless, the general mechanism of adaptation and survival under these conditions are not elucidated yet.

Within its ecological habitat and food chain, *V. parahaemolyticus* encounters changing temperature conditions. These temperature shifts will result in metabolic changes. A cold shock resulting from a rapid downshift of the temperature, e.g. changing water temperatures or storage on ice, alters bacterial gene expression [10–13]. However the expression of *V. parahaemolyticus* resulting from cold shock is still poorly understood. The cold-induced gene expression profile of a clinical *V. parahaemolyticus* strain at 10 °C has been examined by Yang et al. [13] in a time course analysis. Significant differential expression of almost 13 % of genes (*n* = 619) investigated, was found.

Temperatures in the marine habitat of *V. parahaemolyticus* usually do not exceed 25 °C. In *V. parahaemolyticus* several stress proteins, e.g. heat shock protein (*hsp*) families such as Hsp60 (GroEL and GroES) and as Hsp70 (DnaJ, DnaK, GrpE) are produced in response to elevated temperatures [14, 15]. In general those proteins are made in substantial amounts acting as chaperones, protecting cells from heat dependent denaturation [16–18]. In *V. parahaemolyticus* especially Hsp60 family proteins serve as general stress proteins and are found in several cell compartments and in substantial amounts [19].

In addition, changing temperature conditions can affect the pathogenicity of *V. parahaemolyticus* [19]*.* Chiang and Chou [20] demonstrated increased pathogenicity after heat shock response in *V. parahaemolyticus* as elevated toxin expression. Clinical strains alter expression of systems regulating virulence as well as systems indirectly related to host-pathogen attachment such as biofilm production and motility at 37 °C [21]. However, environmental strains did not show this behaviour or exhibit decreased expression of biofilm production or motility related genes at higher temperatures. Sublethal heat shock of *V. parahaemolyticus* resulted in elevated expression levels of the gene encoding the thermostable direct hemolysin (TDH), one of the two prominent toxins enhancing its pathogenicity [19].

The aim of this study was to investigate gene expression profiles of *V. parahaemolyticus* after exposure to 4, 15, 20, 37 and 42 °C. Moreover high regulation clusters e.g. toxins produced in response to temperature changes were to be identified.

## Results and discussion

Understanding temperature-dependent changes in bacterial gene expression patterns is crucial when studying tenacity, invasion, and environmental related viability of bacterial species. Temperature-dependent expression changes as cues for tenacity and persistence within matrixes such as food or hosts and environment has led to genetic approaches defining temperature-induced genes of pathogens [22–24]. However, temperature-dependent induction of genes is an arbitrary parameter because appropriate temperatures for comparison to any other temperature must be assumed. In this study, we investigated temperature-dependent gene expression of *V. parahaemolyticus* in comparable growth phases under different temperatures.

### Validation of microarray results

**Fig. 1:**
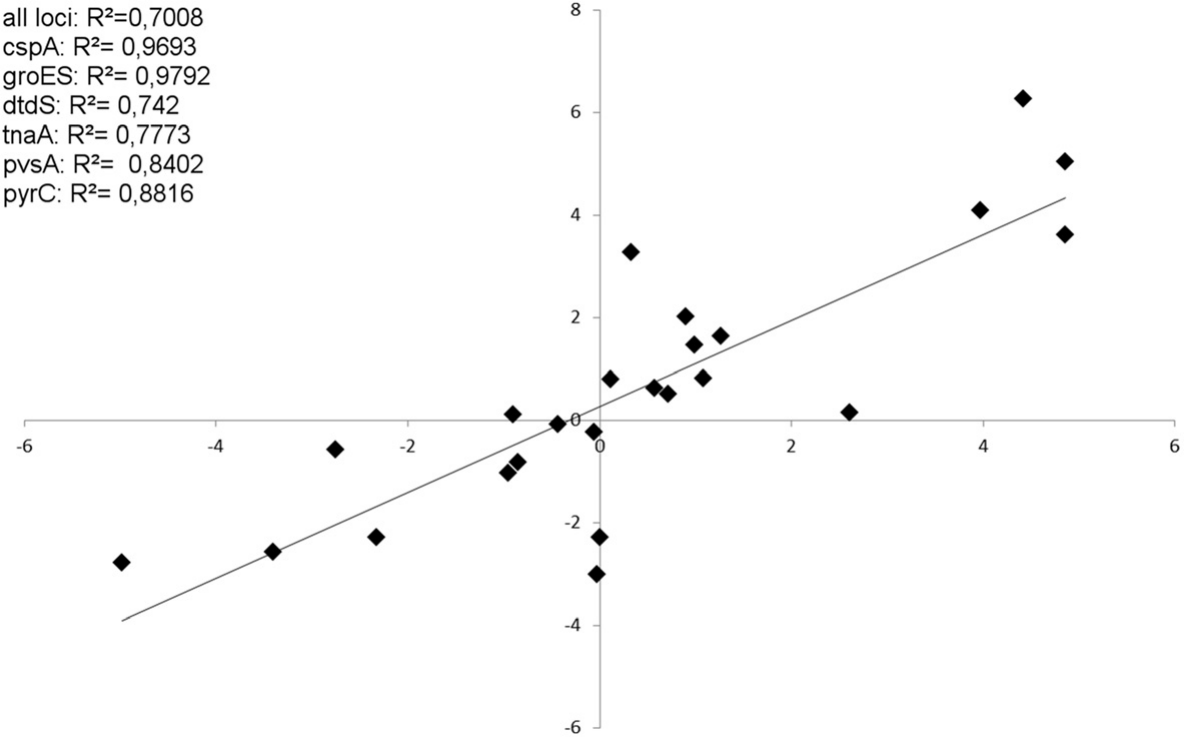
Correlation of microarray and qRT expression of selected genes and quality control. Log_2_ transformed values. R^2^: Coefficient of determination

**Fig. 2:**
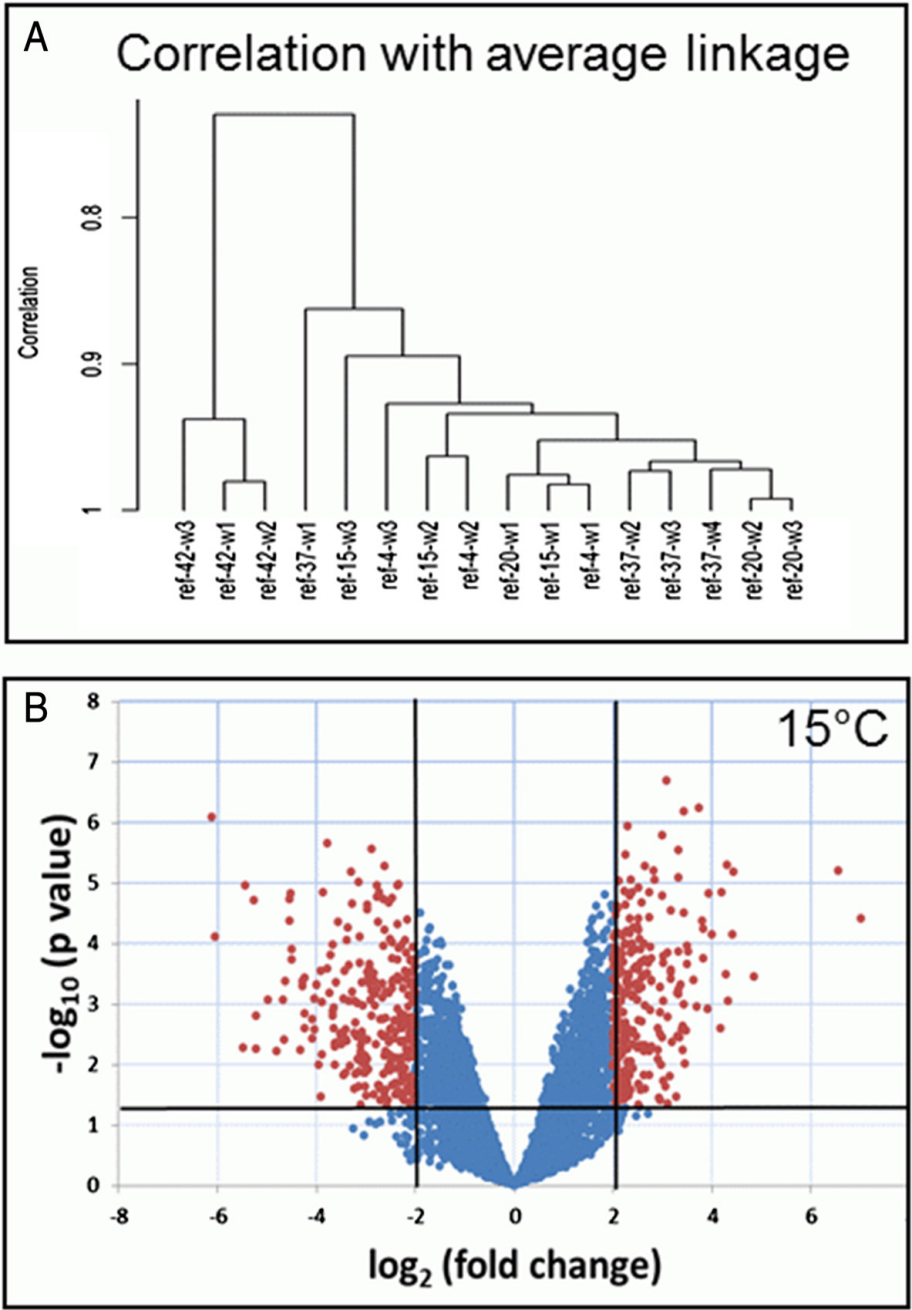
Overview of microarray results. **a** The dendrogram represents the result of hierarchical clustering with euclidean distance measure. The first number in the sample label represents temperature, the second number is the replicate number at given temperature. **b** Volcano plot exemplarily shown for 15 °C data. The x-axis represents the log_2_ of the fold change plotted against the -log_10_ of the adjusted *p*-value. *Red points* indicate the differentially expressed genes with at least 2.0 fold change and statistical significance adjusted p <0.05

To confirm the results of microarray data analysis, a quantitative RT-qPCR was used. Six house-keeping genes were chosen to compare the data of the two techniques, whereof four were applied in the multilocus sequence typing (MLST) scheme of *V. parahaemolyticus* [25]. The house-keeping genes were encoded on both chromosomes, with one exception (*csp*A): *csp*A, *dtd*S, *gro*ES, *pvs*A, *pyr*C and *tna*A. Four additional MLST genes used for normalization: *pvu*A, *dna*E, *rec*A and one locus of the 16S-23S intergenic spacer region. Gene expression values of microarray and RT-qPCR experiments were compared by plotting the log_2_ values of both experiments against each other. An overall positive correlation (R^2^ = 0.7008) between the two techniques could be shown (Fig. 1). The similarity of replicate samples at different temperatures was studied using hierarchical clustering with correlation as the distance measure (Fig. 2a). The samples at 42 °C form the clearest cluster. Samples of 20 and 37 °C cluster according to the temperature. Moreover, volcano plots of microarray data were calculated to visualize the distribution of differentially expressed genes at individual temperatures and to assess quality and comparability of hybridizations (Fig. 2b). Additionally, volcano plots enable the quick identification of expression changes within the gene sets by combination of statistical tests (adjusted *p*-value) and magnitude of changes.

### Gene expression at 4 °C, 15 °C, 20 °C and 42 °C

**Fig. 3:**
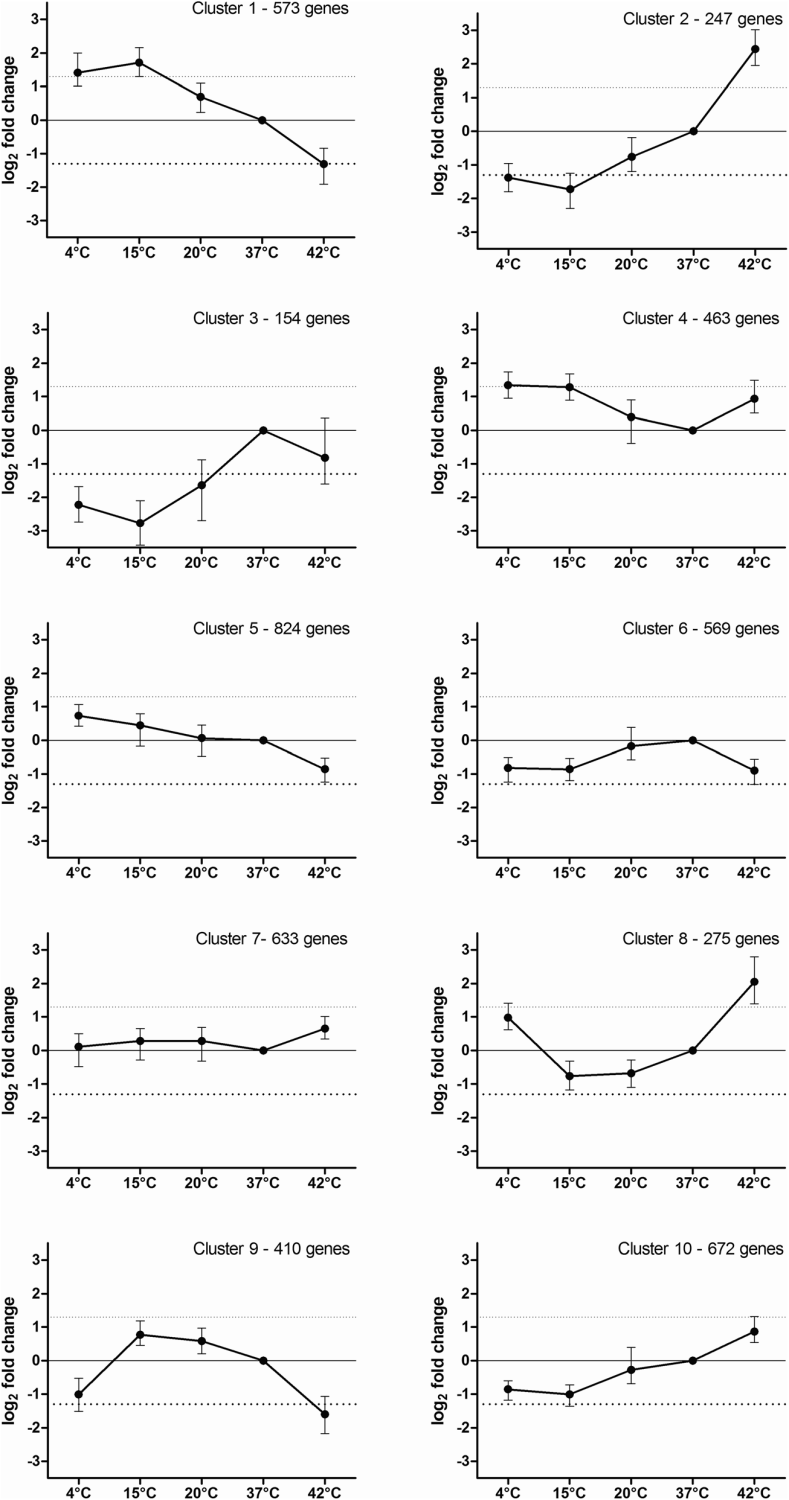
Clustering of genes with similar expression patterns. Ten clusters of similar expressed genes at 4, 15, 20 and 42 °C normalized to 37 °C are shown. The incubation temperatures (x-axis) where plotted against the x-fold gene expression (y-axis) of genes sorted in the particular box. Clustering was performed using *K*-means (genesis)

We compared gene expression patterns within a temperature range of 4 to 42 °C. Additionally, Database for Annotation, Visualization and Integrated Discovery (DAVID) analyses were performed, highlighting regulation of genes connected in metabolic pathways (Additional file 1). Among all conditions, the strongest expression changes (regarding the number of differentially expressed genes and intensity of expression changes) were observed at 15 °C (13.3 % of all genes) and 42 °C (13 % of all genes). Since the highest number of genes with stable expression was found at 37 °C, this temperature was chosen as reference. Genes with an adjusted *p*-value ≤0.05 and an absolute logarithmic fold change ≤±1.5 were considered significantly stable expressed. To demonstrate the temperature-associated differences in gene expression changes, temperature experiments were clustered via *K*-means-clustering (Fig. 3). The *K*-means-clustering arranges genes showing comparable expression under all temperatures investigated. Some genes showing clear up-regulation in both extreme conditions [Fig. 3 - cluster eight, 275 genes including sugar transport system permease (VP0908), *tna*A (VPA0192) a tryptopanase, the putative translation elongation factor G, *ptf*G (VPA0328), the putative phosphatase VPA0505, a putative membrane protein VPA1583] were found to be highly upregulated (>2.0 log_2_). Genes down-regulated at 4 and 42 °C [Fig. 3 - cluster nine, 410 genes including the D-3-phosphoglycerate dehydrogenase VP2593, *eam*A (VP2828) a pore forming protein and glyoxalase I (VP2166)] have been found in high numbers. In addition, there are genes down-regulated across all temperatures (Fig. 3 - cluster three, 154 genes including the putative proteases VP2447, VP2448 and an alcohol dehydrogenase VPA0870). Expression of genes sorted by chromosomes resulted in a higher rate of differentially expressed genes on the small chromosome (chromosome 2) at 15 and 42 °C (Table 1).

**Table 1:** Differentially expressed genes according to encoding chromosome

Incubation temperature	Chr1-up	Chr1-down	Chr2-up	Chr2-down
4 °C	116 (3.77 %)	26 (0.85 %)	35 (2.00 %)	16 (0.92 %)
15 °C	214 (6.96 %)	171 (5.56 %)	147 (8.41 %)	107 (6.12 %)
20 °C	3 (0.10 %)	9 (0.29 %)	2 (0.11 %)	5 (0.29 %)
42 °C	146 (4.75 %)	204 (6.64 %)	187 (10.70 %)	88 (5.04 %)

The numbers of differentially expressed genes are given in total as well as in proportion to the number of genes present on the microarray in brackets. Chr1: genes encoded on chromosome 1 encoding 3080 genes of which 3073 genes were represented on the array. Chr2: genes encoded on chromosome 2 encoding 1752 genes of which 1747 genes were represented on the array. Down: down-regulated genes; up: up-regulated genes

Our analysis identified differentially expressed genes under different temperature conditions. Compared to 37 at 4 °C 4 % (*n* = 193) the genes showed significant expression changes, whereas incubation at 20 °C resulted in a rate of approx. 0.4 % (*n* = 19). At 42 °C, 13 % (*n* = 625) of differentially expressed genes were detected. The highest number of genes regulated, however, was found at 15 °C with 13.3 % (*n* = 638) differentially expressed genes. Incubation at 15 and 42 °C resulted in almost balanced expression patterns regarding the amount of up- and down-regulated genes. At 4 °C, 78 % (*n* = 150) of the significantly differentially expressed genes showed down-regulation, whereas only 22 % (*n* = 43) showed up-regulation. Additional information can be found in Additional file 2.

### Expression of temperature shock response genes

Some gene clusters showed up-regulation of expression under one temperature and down-regulation under another. Chaperone encoding *hsp*70 family genes, such as *dna*K (VP0653), as well as the *hsp*60 family *gro*EL, *gro*ES (VPA0286, VPA0287) showed significant down-regulation of expression at 4, 15 and 20 °C. On the contrary, a strong up-regulation at 42 °C was observed (Additional file 2). Cold shock responding genes, such as *cps*A (VPA1289-1291 and VP1889) as well as a cluster encoding genes classified as ascorbate and phosphotransferase (VPA0229-231) showed significant up-regulation at 4, 15 and 20 °C whereas down-regulation occurred at 42 °C. Yang et al. [13] investigated time dependent behaviour of a clinical *V. parahaemolyticus* strain at cold temperatures. Almost 13 % of genes (619 genes) were differentially expressed at least at one of the three points in time investigated [13]. For metabolism related gene categories down-regulation was dominant over up-regulation due to the generally reduced cellular protein pool resulting from a sudden temperature downshift [11].

These findings are confirmed by our data. Moreover under cold temperatures, non-metabolic functions (cell envelope, transport and binding proteins, regulatory functions, cellular processes and mobile and extra-chromosomal element functions) as well as genes with unknown or unassigned functions showed a more frequently up-regulation than genes related to cell structure and trans-membrane transporting functions (Additional file 1). The cold shock protein/regulator CspA (VPA1289) showed an over 30-fold enhanced transcription. Additionally, an antagonistic regulation of cold and heat shock genes was detected: heat shock genes encoding heat shock proteins (*hsp*), ATP-dependent proteases and chaperons were mainly down-regulated after exposure to 10 °C [11–13]. Our results confirm the findings that metabolism related genes at low temperatures were mainly down-regulated and genes without relation to metabolism or of unknown function were mainly up-regulated. Additionally, antagonistic expression of cold and heat shock genes as well as a strong induction of cold shock proteins at low temperatures was observed for *V. parahaemolyticus* RIMD 2210633 (Additional file 2).

### Gene expression at 4 °C and 15 °C

**Fig. 4:**
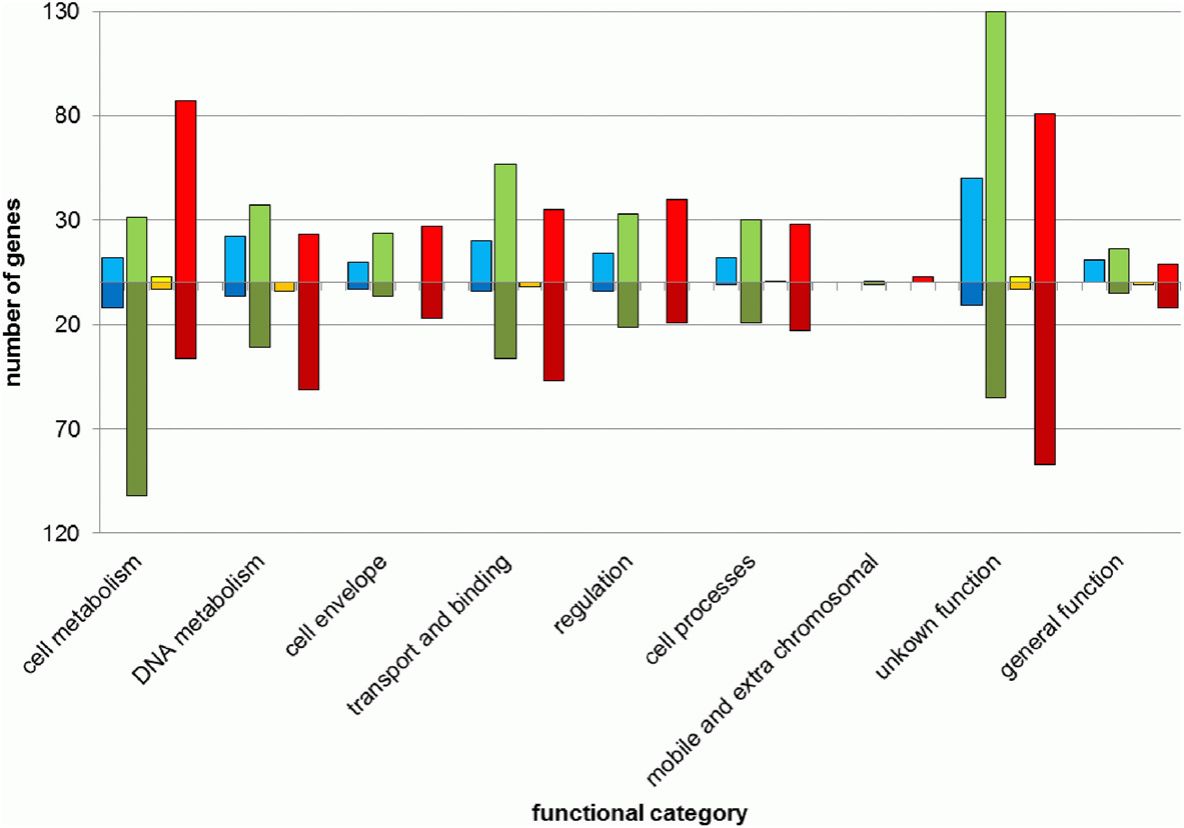
Functional annotation of differentially expressed genes. The amount of genes was plotted according to their function and incubation temperature. Only significantly expressed genes are shown with at least 1.5 log_2_ fold change of expression rate. Normalized incubation temperatures are shown in *blue*, 4 °C; *green*, 15 °C; *yellow*, 20 °C and *red*, 42 °C. *Dark shading* of colour indicates down regulation at the corresponding temperature. All differentially expressed genes with a log_2_ fold change >1.5 and adjusted *p*-value <0.05 in each condition are supplied in Additional file 2

At 4 °C, only 4 % of the genes were differentially expressed. Primarily transcription regulators as well as RNA metabolic process clusters were up-regulated, highlighting the impact of low temperatures (4 °C) on the overall gene expression (Fig. 4). Phadtare et al. [12] described concordant findings in *E. coli*. In our study, at 4 °C mainly genes encoding hypothetical proteins, e.g. VP1888, VP2889, VP3030 and VPA1291 were up-regulated. Additionally, genes of the energy metabolism (VP1381, VP1533, VP2005, VP2666, VP2987, VPA0092) reacted to 4 °C by up-regulation. Especially VP1533, encoding a putative ATPase, is of great importance for energy production using glucose [26]. In particular cold shock proteins were highly expressed (*csp*A VP1889, 4.05 log_2_ fold change). These findings, originally described by Yang et al. [13], were confirmed by our data. However, cold temperatures bias gene expression results due to lower activities of e.g. enzymes [27].

**Fig. 5:**
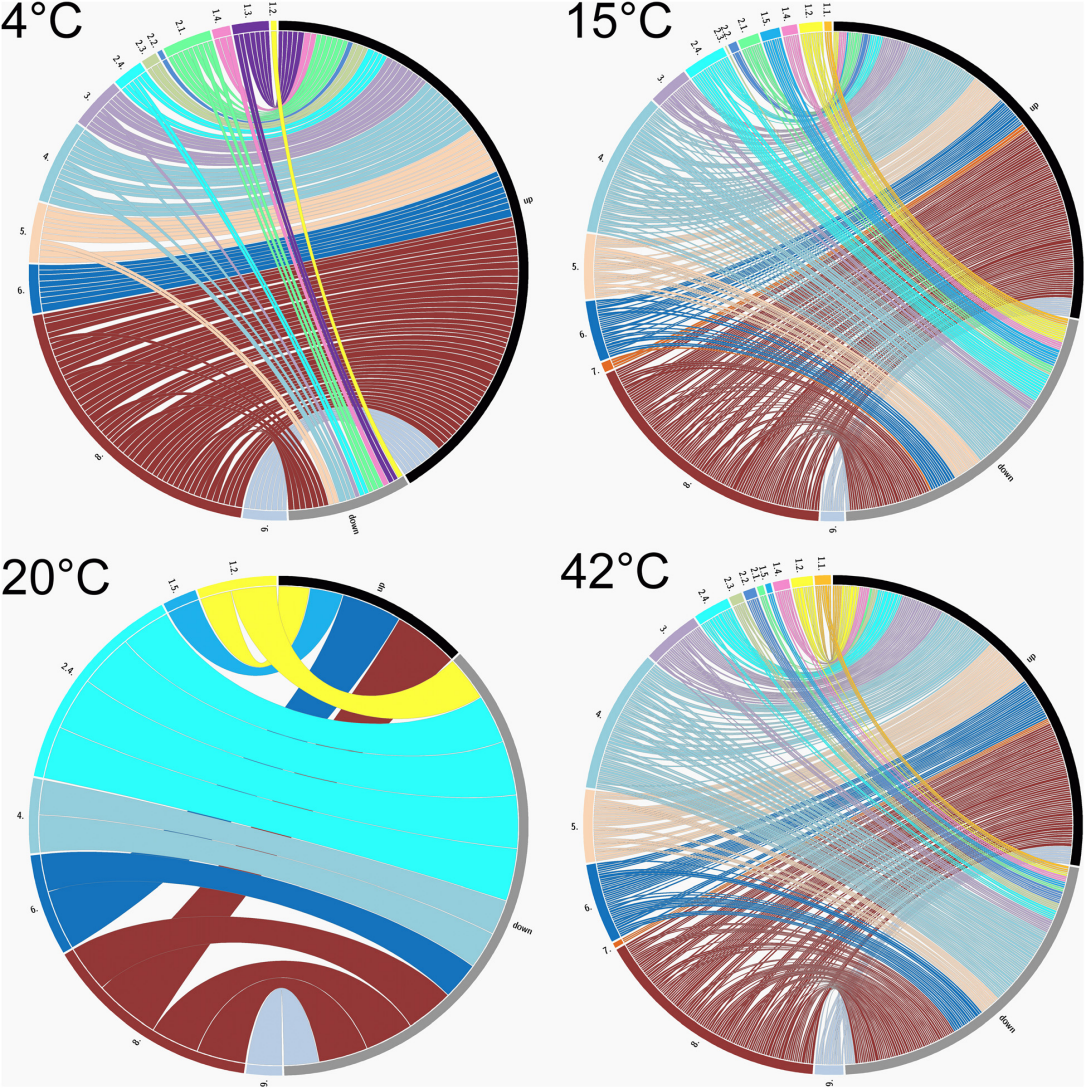
Integrated graphical view of significantly expressed genes of *V. parahaemolyticus* at 4, 15, 20 and 42 °C. The connections show the direction of regulation in each of the functional groups. *Numbers* indicate functional gene groups (*1.1.* Amino acid biosynthesis, *1.2.* Central intermediary metabolism, *1.3.* Energy metabolism, *1.4.* Fatty acid and phospholipid metabolism, *1.5.* Purines, pyrimidines, nucleosides, and nucleotides, *2.1.* DNA metabolism, *2.2.* Transcription, *2.3.* Protein synthesis, *2.4.* Protein fate, *3.* Cell envelope, *4.* Transport and binding proteins, *5.* Regulatory functions, *6.* Cellular processes, *7.* Mobile and extra-chromosomal element functions, *8.* Unknown, *9.* General function). Gene groups were colored according to functional subgroup. The *black bar* highlights up-regulated genes; the *grey bar* indicates down-regulation

The global regulator sigma factor 38, *rpo*S (VP2553) and the osmoregulator *omp*R (VP0154) were up-regulated (3.5 and 4.1×). Sigma factor 38 is one of the most crucial sigma factors under e.g. extreme temperatures [28]. No other sigma factors were up-regulated. Additionally, the tRNA methyltransferase *spo*U (VP0158) described by Persson et al. [29] was up-regulated (4.1×) as well. Persson et al. [29] were not able to detect differences in growth rates of the *E. coli* wild-type and a *spo*U mutant. However, growth temperatures were between 37 and 42 °C in that study. Maybe a particular part of tRNA activation can be triggered by low temperatures. Since none of the known genes related to DNA damage VP2034 (*imu*A), VP2035 (*imu*B), VP2036 (*dna*E2), VP2550 (*rec*A) and VP2945 (*lex*A) were up-regulated, cold induced DNA damaging, triggering the SOS response, appears to be absent. At 4 °C 11 DAVID-gene categories were identified in which a statistically significant number of genes (*n* = 186, *p*-value <0.05) was differentially regulated. Nine of these categories were related to transcription, DNA-binding and regulation of RNA metabolism. The two other categories were related to ABC-transporters or transmembrane domains. The expression of genes organized into functional categories at 4 °C is shown in Fig. 5. The top five up- and down-regulated genes at 4 °C are shown in Table 2.

**Table 2:**
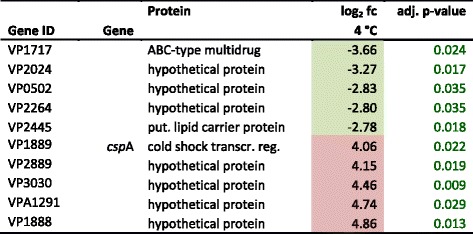
Top 5 up- and down-regulated genes at 4 °C

Coloured boxes highlight at least 1.5 fold differential expression in either direction: red up-regulated, green down-regulated, fc (fold change) is given log_2_ transformed; *transcr. reg.* transcriptional regulator, *put.* putative

At 15 °C a total of 638 genes were differentially expressed (Additional file 2). In addition to *rpo*S, *omp*R and *spo*U, transcriptional regulators VP0034, VP0059, VP0713, VP1391, VP1676, VP1765, VPA0214, VPA1219 and VPA1289 were up-regulated, along with DNA repair VP2943, VPA1393, DNA polymerase III (VP2036), DNA integrase (VP1071). Thus, partially DNA repair has been up-regulated along with protein and peptide secretion and trafficking (VPA1208, VPA1209, VPA1443, VPA 1445). However, no genes related to SOS repair, such as *rec*A (VP2550) and *lex*A (VP2945), or global stress regulators such as *hfq* were up-regulated. These regulators seem of minor concern under these circumstances described. However, strong up-regulation was found for putative regulators such as VP1391 (5×) and VPA1219 (8×).

Especially energy metabolism was down-regulated; out of 75 differentially expressed genes of this category 65 genes (87 %) were repressed (Fig. 4). Genes related to the pentose phosphate pathway, glycolysis and the citric acid cycle were down regulated. We suggest, that production of central energy molecules such as ATP, NADPH and NADH was decreased because of down-regulated expression of corresponding genes. The vast majority of genes showing highest up-regulation, however, were of unknown function (Additional file 2). The putative virulence-associated protein VacB showed highest up-regulation (128×), which has been described to react to environmental signals in *Haemophilus influenza* [30].

Gene expression of functional categories is shown in Fig. 5. In contrast to 4 °C incubation, genes of the amino acid category and *de novo* DNA synthesis were induced at 15 and 42 °C. In total 32 DAVID-gene categories with 475 differentially expressed genes were identified at 15 °C. Ten categories were associated with transport and transporters. Two major groups formed the most important clusters: integral and intrinsic components of the membrane with 69 (11.6 %) genes each. Additionally, nine metabolism related categories were identified.

At 15 °C 32 DAVID-gene categories with 475 genes showed differential regulation. Many of them were connected with membrane maintenance or metabolism. In *E. coli* it could be shown that at 12 °C the membrane composition remains unchanged but enzyme activations are effected [31]. The top five up- and down-regulated genes at 15 °C are shown in Table 3.

**Table 3:**
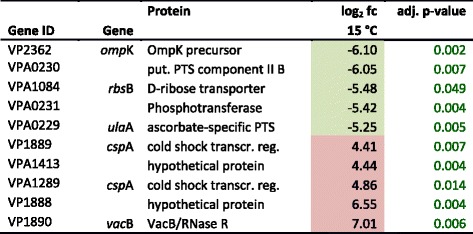
Top 5 up- and down-regulated genes at 15 °C

Coloured boxes highlight at least 1.5 fold differential expression in either direction: red up-regulated, green down-regulated, fc (fold change) is given log_2_ transformed; *PTS* phosphotransferase system; *transcr. reg.* transcriptional regulator, *put.* putative

### Gene expression at 20 °C

At 20 °C, no differential expression of metabolic pathways was detectable. Thus, a range of temperature between 15 and 20 °C seems to describe the (lower) physiological border of the normal condition for the strain investigated. A total of 19 genes was differentially expressed. Gene expression is shown in Fig. 5. At 20 °C solely genes related to categories associated with the degradation of peptides and proteins were identified: ‘peptidase’ (15.8 %), ‘protease’, ‘peptidase activity’ and ‘proteolysis’ (21.1 %), respectively. The top five up- and down-regulated genes at 20 °C are shown in Table 4.

**Table 4:**
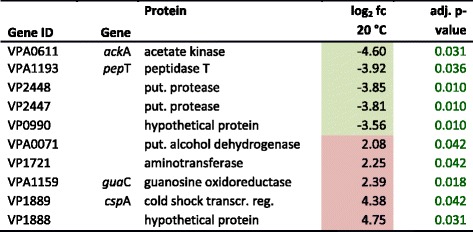
Top 5 up- and down-regulated genes at 20 °C

Coloured boxes highlight at least 1.5 fold differential expression in either direction: red up-regulated, green down-regulated, fc (fold change) is given log_2_ transformed; *transcr. reg.* transcriptional regulator, *put.* putative

### Gene expression at 42 °C

At 42 °C transport and metabolism of carbohydrates-related genes were up-regulated (Additional file 2). Again, a range of temperature between 42 and 37 °C seems to describe the (upper) physiological border for the clinical strain investigated. Interestingly, more genes located on the small chromosome were differentially expressed during incubation at all temperatures. Especially, at 42 °C almost twice as many small chromosome genes were differentially expressed. The higher intensity of expression changes in genes located on the small chromosome compared to genes located on the large chromosome can be explained by the higher number of genes related to transcriptional regulation and transport of various substances being located on the small chromosome [32]. Thus, most genes related to environmental stress response are encoded on the small chromosome.

Primarily, genes classified as ‘cell metabolism’ along with the genes classified as ‘unknown’, reacted to the temperature upshift. Altogether, the expression of 625 genes was differentially expressed at 42 °C. Expression of categorized genes is shown in Fig. 5. Out of the 87 ‘cell metabolism’ genes, 55 % (*n* = 48) were classified as ‘energy metabolism’ related genes.

A wide spectrum of genes was affected. For example, genes associated with amino acid and amine synthesis (pyruvate family) were induced, whereas genes related to histidine (VP1137), serine (VP1324, VP1629, VP2593) and aromatic amino acid (VP2744, VP3065) families were down-regulated (Additional file 1). Out of 55 energy metabolism related genes only six were down-regulated in expression. Particularly, genes of electron transfer (VP1161, VPA0643, VPA0949, VPA1428), biosynthesis of polyamines (VPA0169, VPA0170, VPA1635) and degradation of fatty acids as well as fermentation (VP1647, VP2543, VPA0478, VPA0502, VPA1416) were up-regulated at 42 °C. Moreover especially sugar metabolism (VP1303, VP2397, VP2398, VP2400, VPA1674, VPA1675, VPA1700, VPA1706) was affected (Additional file 1). Genes involved in arabinose (VPA 1671–1678), mannose and glucoronate (VPA1702-1709) metabolism and transport were up-regulated. Additionally heat protection protein encoding genes such as *gro*EL, *gro*ES were induced. Reactions of heat shock proteins such as GroEL/GroES, are in concordance with data described by Wong et al. [19].

At 42 °C, 38 DAVID-gene categories with a total of 423 differentially expressed genes were identified (Additional file 1). Amongst others, nine categories were related to cell-motion (flagella), eight categories to metabolic processes and six categories to RNA, DNA and transcription. Additionally, three categories were associated with homeostasis (ion, cation, chemical) and two categories with iron-siderophores and transport of siderophores. A distinct cluster on the second chromosome encoding the genes VPA0915-1042 (‘cellular processes’: *n* = 23, ‘energy metabolism’: *n* = 18, ‘transport and binding’: *n* = 14, ‘regulatory functions’: *n* = 14 and ‘unknown’: *n* = 36) showed up-regulation at 42 °C. The top five up- and down-regulated genes at 42 °C are shown in Table 5.

**Table 5:**
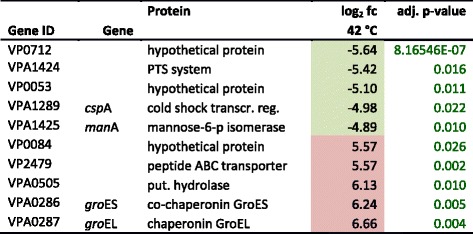
Top 5 up- and down-regulated genes at 42 °C

Coloured boxes highlight at least 1.5 fold differential expression in either direction: red up-regulated, green down-regulated, fc (fold change) is given log_2_ transformed; *transcr. reg.* transcriptional regulator, *put.* putative

However, no prior studies about genome wide gene expression responses exist for the temperatures investigated in this study.

### Temperature dependent expression of virulence genes

Virulence genes in total showed no significant expression changes under different temperatures (Additional file 2). The expression of *tdh* was not significantly influenced by temperature changes, even though slight activation (2.1 log_2_ fold change) was observed at 15 °C. A putative haemolysin encoding gene (VP3048), was up-regulated at 4 and 15 °C. This effect was described by Yang et al. [13], reporting an induction of this putative haemolysin after cold shock. The most prominent haemolysin *tdh*, however, was not significantly up-regulated (Additional file 2). The associated regulator *opa*R which recently has been shown to repress expression of T6SS in *V. parahaemolyticus* is down-regulated at 42 °C [33]. We found that, genes located within the virulence pathogenicity island 7 (VPa-7) encoded on the small chromosome, VPA1312-1396 showed no reaction to thermal stimulations (Additional file 2). However, since the energy metabolism was affected especially and mostly at cold temperatures, reduced classical virulence or changed expression rates were to be expected [34].

Virulence associated genes in general (*tdh*1, *tdh*2, *tox*R, *tox*S, *vop*C, T6SS-1: VP1386-1420), remained unaffected by heat or cold stress (Additional file 2). The T3SS-1 was found down-regulated at 15 °C for *nos*A (VP1697) and up-regulated for the putative chaperone VP1687 at 42 °C. However, the T6SS-1 located on chromosome 1 showed up-regulation at 42 °C. This was to be expected since the T6SS-1 system reacts to warm climate in *V. parahaemolyticus* as described by Salomon et al. [35]. The cold shock gene *csp*A was down-regulated, whereas heat shock genes encoding chaperones and protection via sugar metabolites were induced [13].

## Conclusions

Based on our data, the optimal temperature range of the clinical *V. parahaemolyticus* strain investigated is between 20 and 37 °C, since most of the genes were transcribed at a rather constant level.

Finally, it could be shown that the classical pathogenicity markers, T3SSs as well as T6SSs were not up-regulated in response to thermal changes. However, large proportions (~30 %) of the differentially expressed genes are of unknown function. Summarized, this study successfully demonstrated that genome-wide gene expression changes in *V. parahaemolyticus* occur at 4, 15, 20, and 42 °C.

## Methods

### Bacterial strains

*V. parahaemolyticus* RIMD2210633 was isolated from a patient suffering from diarrhoea in Japan in 1996 [32]. This strain harbours the *tdh* gene, lacks the *trh* gene and belongs to serotype O3:K6 [36]. This serotype has been detected in clinical as well as in environmental marine samples [37]. The strain has been sequenced by Makino et al. [32].

Prior use, the strain was stored in cryovials at −80 °C (Cryobank; Mast Diagnostica, Bootle, England). For initial growth, cells were grown using a rotary shaker (Unimax 1010 and Incubator 1000; Heidolph, Schwabach, Germany) in alkaline peptone water (APW; 0.3 % Yeast-Extract, 1 % Peptone, 2 % NaCl; pH 8.6) at 37 °C overnight. A 2 ml aliquot of the resulting culture was diluted to a total volume of 25 ml using APW and grown to an A_600 nm_ of 0.6. Cultures were grown at 37 °C for 3.5 h in order to generate exponential phase cultures. After appropriate dilutions the A_600_ was analysed again and aliquots consisting of 10^8^ to 10^9^ *V. parahaemolyticus* cells were transferred to 15 ml Falcon tubes, placed in a thermal mixer (Thermomixer comfort; Eppendorf, Hamburg, Germany) and incubated at different temperatures (42, 37 and 20 °C) for 30 min. For stressing the cells at 4 and 15 °C the entire incubation unit was placed in a conditioning cabinet (Rubarth Apparate, Laatzen, Germany) and bacteria were incubated at these temperatures for 30 min.

### RNA preparation and reverse transcription for qPCR investigation

The cultures were centrifuged (2 min, 8000 × g) and the supernatant was discarded. The pellet was immediately resuspended in 1.5 ml RNAprotect Bacteria Reagent (Qiagen, Hilden, Germany) to minimize RNA degradation. Total RNA was isolated using the peqGold Bacterial RNA Kit (Peqlab, Erlangen, Germany). The obtained RNA was eluted into 43 μl of DEPC-treated, DNase- and RNase-free water (Carl Roth, Karlsruhe, Germany). Samples were then treated with DNase I along with Ribolock, an RNase-A, −B and -C inhibitor (Fermentas, Vilnius, Lithuania). RNA quantity was measured by spectrophotometry. RNA quality of each sample was monitored via gel electrophoresis. Additionally, the RNA quality was assessed using the Agilent RNA 6000 Nano Kit on a 2100 Bioanalyzer (Agilent, Santa Clara, US).

### Fluorescence-labeled cRNA generation for the microarray

Prior to labelling, the RNA was initially transcribed in cDNA. Briefly, 200 ng of RNA were linear amplified using the full spectrum MultiStart primer (Biocat, Heidelberg, Germany) and Moloney murine leukemia virus reverse transcriptase (Agilent). The amplification was performed at 40 °C for 2 h followed by 65 °C for 15 min and stored at 4 °C. The amplified cDNA, the full spectrum MultiStart primer and T7 RNA polymerase were used along with Cyanine 3-CTP (Agilent) generating labelled cRNA. Labeling was performed using the Quick Amp Labeling Kit (Agilent). The labeled cRNA was purified using the Qiagen RNeasy Mini Kit (Qiagen). A 3 μl-aliquot was used for quality control. Experiments were performed using Agilent custom 8 × 15 k arrays (Agilent). The microarray field covers 99.75 % of all *V. parahaemolyticus* genes. In total, 3073 out of 3080 genes encoded on chromosome 1 and 1747 out of 1752 genes located on chromosome 2, are included. Each gene is represented by 1 to 10 probes (mean 3.15 probes per gene). Each probe consists of a 60mer located preferentially at the 3’ terminus of the corresponding gene. The probe design was performed with the eArray Software a web-based Agilent application basing on the genome sequence of *V. parahaemolyticus* RIMD 2210633 (http://www.ncbi.nlm.nih.gov/genome/691?genome_assembly_id=167995). The cRNA samples were then hybridized to an individual microarray field.

### Microarray hybridization and post hybridisation washing

For hybridizations on the microarray, three replicates of independently grown bacterial cultures were prepared for each temperature condition, for 37 °C four replicates were used. Accordingly, three individually labeled cRNA sets were prepared for each temperature other than 37 °C. Finally, 600 ng of the labeled and linear amplified cRNA was fragmented, added to 25 μl hybridization buffer mix of which a 20 μl aliquot (480 ng) was loaded on a microarray in a hybridization chamber (Biometra, Goettingen, Germany). The one-channel hybridization was performed at 65 °C for 17 h and 10 rpm.

Washing of the slides was performed using preheated washing buffer (Gene expression wash buffer kit, Agilent). First the chamber was rinsed with washing buffer. Then the slides were washed once followed by a second washing step using washing buffer containing 0.01 % Triton X-102 (Agilent). The slides were dried using acetonitrile.

### Data handling and microarray analysis

Scanning was carried out using the Agilent G2565CA scanner with a resolution of 5 μm. After scanning, tiff-files were analysed and raw data was extracted using Feature Extraction Software (Agilent). Data processing was performed using Bioconductor V 2.12 package of the software R. At first, background corrected spot intensities (signal gProcessedSignal in the Agilent protocol GE1_107_Sep09) were retrieved and bad quality spots were removed using the outlier detection flags of the Agilent protocol. Further, the signal values were normalized using quantile normalization and log_2_ transformed [38]. Linear modelling and empirical Bayes methods, implemented in the R package Limma [39], were used to detect the differentially expressed genes between two groups, in this case, the control and treatment sample groups. Raw *p*-values were adjusted using the Benjamini and Hochberg multiple adjustment method [40]. Genes with an adjusted *p*-value ≤0.05 and an absolute logarithmic fold change ≥1.5 were considered significantly differentially induced, while genes with an absolute logarithmic fold change ≤ −1.5 were considered repressed. Annotation of genes was performed according to Yang et al. [13] and updated using two new gene entries at NCBI (http://www.ncbi.nlm.nih.gov/gene), KEGG (http://www.genome.jp/kegg/) and Gene Ontology (http://www.geneontology.org/).

Finally, enriched terms were searched in annotations as well as functional-related gene categories using the Database for Annotation, Visualization and Integrated Discovery (DAVID V 6.7, Fisher exact test) [41, 42]. The gene lists generated via DAVID enable to highlight gene sets which show a higher proportion of differentially expressed genes compared to other categories. This eases identification of pertinent biological processes to the according temperature. The identified categories are presented in the Additional file 1. *K*-means clustering of genes with similar gene expression was performed using Genesis V 1.7.6 [43]. Heat maps were generated using BioNumerics V 6.01 (Applied Math, St. Martens-Latem, Belgium). Volcano plots were generated via GraphPad V 5.04, (GraphPad, San Diego, US). Integrated graphical views were generated using Circos plot [44]. The transcriptomics data were supplied as experiment GSE60815 at Gene Expression Omnibus according to MIAME regulations. All differentially expressed genes with a log_2_ fold change >1.5 and adjusted *p*-value <0.05 of each condition are supplied in Additional file 2.

### qRT-PCR

For generating cDNA, a 1 μg RNA aliquot was used and reversely transcribed by the RevertAid Premium First Strand cDNA Synthesis Kit and random hexamer primers according to the manufacturer’s instructions (Fermentas). Additionally, 1 μg of total RNA was used as RT- negative control following the same protocol with additional reaction buffer instead of the enzyme mix. Resulting cDNAs as well as RT-negative controls were diluted 1:50 in DNase- and RNase-free water. 1 μl of each sample was used for qRT-PCR.

Specific oligonucleotide primer pairs were used for PCR (Table 6). New primers or new primer pairs were designed with Primer3 software (http://frodo.wi.mit.edu/) and synthesized (Metabion, Martinsried, Germany). The amounts of cDNA of all genes were determined by qRT-PCR assays in 12.5 μl reaction volume. Conditions for the reactions were: 6.25 μl of 2× SsoFast Eva Green Supermix (BioRad, Hercules, US), 0.5 μM of each primer, 1 μl of cDNA; 1 × 95 °C for 3 min, 45 × 95 °C for 10 s and 57 °C for 15 s in a BioRad C1000 cycler with an CFX96 optical head. Validation of specific products was done via melting curve analysis, consisting of an initial heating at 95 °C for 10 s, followed by a stepwise temperature increase from 68 to 88 °C with an increment of 0.2 °C for 5 s. Threshold cycle values were calculated via regression analysis using CFX manager V 2.0 (BioRad). Differentially expressed genes were identified and analysed with the option ‘gene study’ of CFX manager software. The genes *pvu*A, *dna*E, *rec*A and a locus of the 16S-23S intergenic spacer region (1623S) were used for normalization via ΔΔC(q)-method.

**Table 6:** qRT-PCR primers

Gene	ID	Sequence 5′ to 3′	Size [bp]	Reference
1623S	bp 134385	GCTGACAAAACAACAATTTATTGTT	170	[45]
	to 135166^a^	GGAGTTTCGAGTTGATGAAC		
*gro*ES	VP2852	TATTCAACGATCGCCATGAT	108	This study
		TGGTGACACCGTTATCTTCG		
*csp*A	VPA1289	TATCGTTGCTGACGGTTTCA	90	This study
		TCAGTCGCTTGAGGACCTTT		
*pvs*A	VPA1658	GGACCTCCACGTCGTTCTTA	112	This study
		GGGATTGAAGACATCGCACT		
*pvu*A	VPA1656	GCTGTCGATGCTTGATCGTA	107	This study
		GTGGAATCGGTTTGGTCACT		
*rec*A	VP2550	GAAACCATTTCAACGGGTTC	139	[25]
		GTGCAGCAGCGATAAGCTC		This study
*dna*E	VP2303	GATTACCGCTTTCGCCG	140	[25]
		GTGTATCCATGCCCGATTTC		This study
*dtd*S	VPA1508	TGGCCATAACGACATTCTGA	124	[25]
		TTCGTGACCGACAACCATAG		This study
*pyr*C	VPA0408	AGCAACCGGTAAAATTGTCG	142	**[** 25 **]**
		TCCATGAACCAAAAGCAACA		This study
*tna*A	VPA0192	TGTACGAAATTGCCACCAAA	103	[25]
		TCAGCGTAACCTTCTTCACG		This study

^a^ 16S-23S intergenic spacer region encoded on chromosome 2

## Availability of supporting data

The transcriptomics data were supplied as experiment GSE60815 at Gene Expression Omnibus according to MIAME regulations. Available at: http://www.ncbi.nlm.nih.gov/geo/query/acc.cgi?acc=GSE60815.

## Additional files

Additional file 1:
**DAVID categories.** Additional file includes DAVID data for all temperatures. (XLSX 25 kb) Additional file 2:
**Differentially expressed genes with log**_**2**_ **fold change >1.5 and adjusted** ***p*** **-value <0.05 in each condition.** Additional file includes the homolog and antagonistic reacting genes. (XLSX 192 kb)

## Abbreviations

DAVID: Database for Annotation, Visualization and Integrated Discovery
T3SS-1: Type three secretion system one
T6SS: Type six secretion system
TDH: Thermostable direct hemolysin
TRH: Thermostable direct hemolysin related hemolysin
